# Assessment of Bidirectional Relationships Between Polycystic Ovary Syndrome and Periodontitis: Insights From a Mendelian Randomization Analysis

**DOI:** 10.3389/fgene.2021.644101

**Published:** 2021-03-26

**Authors:** Pengfei Wu, Xinghao Zhang, Ping Zhou, Wan Zhang, Danyang Li, Mingming Lv, Xiaoyao Liao

**Affiliations:** ^1^Hunan Key Laboratory of Animal Models for Human Diseases, Department of Laboratory Animals, Third Xiangya Hospital, Central South University, Changsha, China; ^2^Hunan Key Laboratory of Medical Genetics, School of Life Sciences, Center for Medical Genetics, Central South University, Changsha, China; ^3^Department of Ultrasound, Third Xiangya Hospital, Central South University, Changsha, China; ^4^Department of Biology, College of Arts and Sciences, Boston University, Boston, MA, United States; ^5^Department of Oral Maxillofacial-Head Neck Oncology, College of Stomatology, Shanghai Ninth People’s Hospital, Shanghai Jiao Tong University School of Medicine, Shanghai, China; ^6^Shanghai Key Laboratory of Stomatology, National Clinical Research Center for Oral Diseases, Shanghai Research Institute of Stomatology, Shanghai, China; ^7^School of Medicine, Dentistry and Nursing, University of Glasgow, Glasgow, United Kingdom

**Keywords:** periodontitis, polycystic ovary syndrome, Mendelian randomization, causal inference, genetic epidemiology

## Abstract

**Background:**

Observational studies have indicated an association between polycystic ovary syndrome (PCOS) and periodontitis, but it is unclear whether the association is cofounded or causal. We conducted a two-sample Mendelian randomization (MR) study to investigate the bidirectional relationship between genetically predicted PCOS and periodontitis.

**Methods:**

From two genome-wide association studies we selected 13 and 7 single nucleotide polymorphisms associated with PCOS and periodontitis, respectively, as instrumental variables. We utilized publicly shared summary-level statistics from European-ancestry cohorts. To explore the causal effect of PCOS on periodontitis, 12,289 cases of periodontitis and 22,326 controls were incorporated, while 4,890 cases of PCOS and 20,405 controls in the reverse MR. Inverse-variance weighted method was employed in the primary MR analysis and multiple sensitivity analyses were implemented.

**Results:**

Genetically determined PCOS was not causally associated with risk of periodontitis (odds ratio 0.97; 95% confidence interval 0.88–1.06; *P* = 0.50) per one-unit increase in the log-odds ratio of periodontitis. Similarly, no causal effect of periodontitis on PCOS was shown with the odds ratio for PCOS was 1.17 (95% confidence interval 0.91–1.49; *P* = 0.21) per one-unit increase in the log-odds ratio of periodontitis. Consistent results were yielded via additional MR methods. Sensitivity analyses demonstrated no presence of horizontal pleiotropy or heterogeneity.

**Conclusion:**

The bidirectional MR study couldn’t provide convincing evidence for the causal relationship between genetic liability to PCOS and periodontitis in the Europeans. Triangulating evidence across further observational and genetic-epidemiological studies is necessary.

## Introduction

Polycystic ovary syndrome (PCOS) is a metabolic and hormonal disorder, which is prevalent in women of reproductive ages. About 15–20% premenopausal women are afflicted with PCOS in Europe (Sirmans and Pate, 2013; Escobar-Morreale, 2018) according to the Rotterdam criteria (Rotterdam ESHRE Group, 2004). PCOS is characterized by hyperandrogenism (HA), ovulatory dysfunction (OD), and polycystic ovarian morphology (PCOM). Unfavorable metabolic conditions, such as insulin resistance and endocrine-reproductive comorbidities, are commonly involved in the pathophysiology of PCOS (Azziz et al., 2019). As for the etiology, the complex interplay of genetic and environmental elements has been well recognized (Yu et al., 2018). However, the comprehensive links between PCOS and its downstream traits are waiting to be explored, as well as their underlying factors.

Periodontitis has posed an increasingly substantial burden on public health (Chaffee et al., 2020; Eke et al., 2020). Periodontitis features deterioration of local periodontal tissues, progressive destruction of alveolar bone and supporting ligament, and ultimately could result in tooth loss. Inadequate oral hygiene and microbe plaque accrual is known as initiation factors. Meanwhile, host susceptibilities to periodontopathic germs and inflammatory response are partly determined genetically; several loci have been identified in suggestive association with periodontitis (Divaris et al., 2013; Offenbacher et al., 2016; Munz et al., 2017, 2019; Kurushima et al., 2019). Moreover, periodontitis has much wider implications in multiple systems (Czesnikiewicz-Guzik et al., 2019; Bae and Lee, 2020; Sun et al., 2020) beyond oral health alone, albeit the underpinning is largely unidentified.

Emerging studies have proposed the association between PCOS and periodontitis (Kellesarian et al., 2017; Machado et al., 2020; Marquez-Arrico et al., 2020). As an essential parameter in the diagnosis of periodontitis, periodontal probing depth (PPD) was higher in PCOS in one recent cross-sectional research (Isik et al., 2020). The latest meta-analysis (Machado et al., 2020) suggested that patients with PCOS were at 28% higher odds of having periodontitis, and periodontitis increased the odds of PCOS by 46% (both *P* < 0.001). Notably, current evidence was mostly drawn from case-control or cohort studies, while high-quality studies like randomized controlled trails were scarce. Hence, effect estimates were prone to bias. Due to inherent weaknesses of traditional observational designs, we could not figure out whether the effects were causative. The direction of causality which was informative of risk factors and prevention strategies in the bidirectional association between PCOS and periodontitis, if existed, remains unknown.

Mendelian randomization (MR) is a powerful genetic-epidemiological tool to strengthen the causal inference and give the robust estimate (Burgess et al., 2019; Czesnikiewicz-Guzik et al., 2019; Bae and Lee, 2020; Sun et al., 2020), especially when well-powered randomized clinical trials are faced with financial challenges and ethical dilemmas. MR studies employs single nucleotide polymorphisms (SNPs) identified from the genome-wide association study (GWAS) as instrumental variables. MR design is supposed to give evidence whose strength is comparable to that of the meta-analysis (Davies et al., 2018). Therefore, we performed a bidirectional MR study to investigate the possible causal role for PCOS on periodontitis, along with the reverse causal effect of periodontitis on PCOS.

## Materials and Methods

### Overall Study Design

**FIGURE 1:**
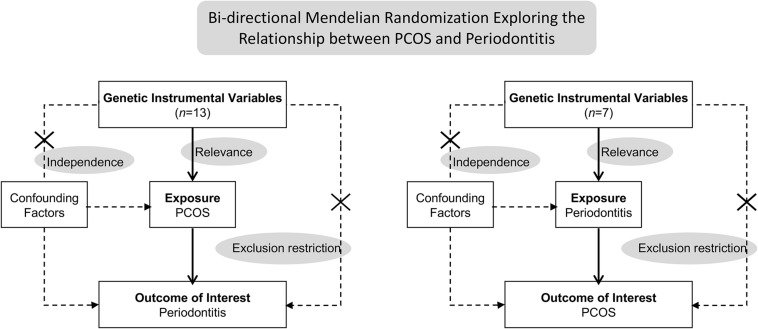
Schematics for the bidirectional Mendelian randomization design. Mendelian randomization requires valid genetic instrumental variants satisfying three assumptions. PCOS, polycystic ovary syndrome.

This bidirectional MR study was undertaken in a framework as delineated in Figure 1. Causal effects of PCOS on periodontitis and the reverse causation were investigated separately. Three key assumptions underlie the MR analysis (Burgess et al., 2019). Firstly, the relevance assumption was met, considering genetic variants associated with exposures of interest were identified in sufficiently large-sample GWAS. Secondly, the independence assumption was validated. Mendel’s laws make perfect randomization, that is, randomized segregation and independent assortment of alleles during gamete formation far precedes the onset of oucome diseases concerned. Hence, instrumental SNPs rarely links to confounders which are commonly involved in the traditional observation study examining the exposure-outcome relationship. Lastly, the exclusion-restriction assumption requires that instrumental variables exert influences on the outcome via no other pathways than the exposure, also known as pleiotropic effects. We have examined the potential pleiotropy through multiple sensitivity analyses. This MR study was conducted using publicly shared datasets, and the approval by concerned ethical committee and consent from all participants were obtained in the original GWAS studies and their contributing cohorts. Additional ethic statement or consent was not required.

### Summary Statistics for PCOS

Day et al. (2018, 2019) has conducted the largest GWAS meta-analysis of PCOS in 10,074 cases and 103,164 controls of European ancestry and identified 14 SNPs at genome-wide significance (*P* < 5 × 10^–8^). Diagnosis of PCOS is based on National Institutes of Health criteria (Carmina, 2004), Rotterdam criteria (Rotterdam ESHRE Group, 2004), or self-report questionnaire (Day et al., 2015). Presence of both OD and HA satisfies the National Institutes of Health criteria, while Rotterdam criteria incorporates PCOM and requires two out of three principal traits to be met. Self-reported diagnosis was used in the 23 and Me (Mountain View, CA, United States) cohort, and due to the data shared policy, summary-level statistics from 4,890 cases of PCOS and 20,405 controls excluding this cohort were available. Nevertheless, 14 genome-wide significant loci manifested negligible heterogeneity in the effect direction and magnitude, after examining the odds ratio for PCOS as a function of diagnostic criteria. Shared genetic architecture across three diagnostic criteria was elaborated in the GWAS (Day et al., 2018). Hence, we utilized summary statistics for PCOS derived from as large sample fulfilling either criterion.

In the MR analysis exploring causal effects of PCOS on periodontitis, 13 SNPs were selected as instrumental variables (Supplementary Table 1). SNPs with minor allele frequency less than 1% or Hardy-Weinberg equilibrium test *P*-value less than 0.0001 will not be considered. Palindromic alleles with minor allele frequency above 0.45 was also excluded due to the ambiguous strand aligning issue. One such variant (rs853854, A/T, allele frequency, 0.499/0.501) reported in the original GWAS, hence was not selected as instrumental variables for PCOS. Linkage disequilibrium (threshold set at *R*^2^ > 0.01, within 1 Mb window, EUR panel of 1,000 Genomes Project Phase 3) was examined (Myers et al., 2020) and the variant with the lowest *P*-value at each locus was retained. Look-up of potential pleiotropic associations (Supplementary Table 2) was performed in the GWAS Catalog (Buniello et al., 2019). Proportion of variance explained was calculated using the formula 2 × MAF × (1 – MAF) × Beta^2^, where MAF was the minor allele frequency, Beta represented the estimated genetic effect on the risk of PCOS. Total variance explained by instrumental SNPs for PCOS approximated 6.2% (Supplementary Table 3). The strength of each SNP was assessed by *F*-statistic using the formula *R*^2^(*N* – 2)/(1 – *R*^2^), where *R*^2^ was the proportion of variance explained, *N* was the total sample size. *F*-statistic for individual variant ranged from 30.8 to 57.6; therefore, none was weak instrument (*F* < 10). GWAS results for PCOS are publicly available from Apollo^1^. Effect size has been adjusted for age and presented as beta (log-odds) per additional effect allele.

### Summary Statistics for Periodontitis

Summary-level data for periodontitis were obtained from the newly released GWAS (Shungin et al., 2019). Totally, this GWAS incorporated 12,289 cases and 22,326 controls of European-ancestry from seven contributing cohorts in the Gene-Lifestyle Interactions in Dental Endpoints consortium. The clinical diagnostic criteria by the Centers for Disease Control and Prevention/American Academy of Periodontology (Page and Eke, 2007) and self-reported diagnosis from the Women’s Health Study at Brigham and Women’s Hospital (Yu et al., 2018) were primarily adopted, whereas additional inclusion criteria were defined as one of the following conditions, two or more tooth surfaces with PPD ≥ 5 mm, or four or more with PPD ≥ 4 mm, two or more tooth surfaces with PPD ≥ 5.5 mm or dental records of “gum surgery”.

For the MR analysis of periodontitis on PCOS, seven instrumental SNPs associated with periodontitis (*P* < 5 × 10^–6^) were selected (Supplementary Table 4) since no genome-wide significant loci were identified in the European-ancestry GWAS (Shungin et al., 2019). No pleiotropic associations were identified through look-up in the GWAS Catalog (Supplementary Table 5). Considering allele frequency variable has been removed to prevent re-identification of individuals in the shared dataset, we utilized the reference minor allele frequency from 1,000 Genomes European panel to calculate *F*-statistic. Although a more liberal threshold (*P* < 5 × 10^–6^) was adopted, there was no evidence of the existence of weak instrument (Supplementary Table 6). Initially, 20 SNPs reaching an arbitrary threshold for suggestive association (*P* < 5 × 10^–6^) were retrieved from the summary-level dataset. Criteria of instrumental SNPs for periodotitis, like minor allele frequency and linkage disequilibrium, were set similar to the criteria of instrumental SNPs for PCOS. Linkage disequilibrium was examined, and nine SNPs with the lowest *P*-value at each locus were kept. Two SNPs were further omitted, for whom or their proxies (*R*^2^ > 0.8), corresponding statistics were not present in the PCOS dataset. Effect estimates denote log-odds of periodontitis given by the additive genetic model, adjusted for age and principal components as covariates. Summary-level statistics for periodontitis can be obtained from the dataset depository, University of Bristol^2^.

### Statistical Analysis

The statistical analysis was performed using the R software, version 3.6.1 (R Foundation for Statistical Computing, Vienna, Austria) and *TwoSample MR* and *MR-PRESSO* packages (Hemani et al., 2018; Verbanck et al., 2018). The inverse variance weighted (IVW) approach was implemented as the primary MR method to yield an overall estimate from multiple instrumental variables (Burgess et al., 2013). Specifically, for each variant SNP*_*k*_*, its genetic effect on the exposure and outcome per additional effect allele, ${\hat{\beta }}_{X_{k}}$ and ${\hat{\beta }}_{Y_{k}}$, and their standard errors ${\hat{\sigma }}_{X_{k}}$ and ${\hat{\sigma }}_{Y_{k}}$, MR causal estimates can be given by the Wald ratio ${\hat{\beta }}_{Y_{k}}\,/\,{\hat{\beta }}_{X_{k}}$ with the standard error ${\hat{\sigma }}_{Y_{k}}\,/\,{\hat{\beta }}_{X_{k}}$. Then an overall causal estimator ${\hat{\beta }}_{\text{IVW}}$ with standard error ${\hat{\sigma }}_{\text{IVW}}$ can be derived as shown below.

β^IVW=Σk⁢β^Xk⁢β^Yk⁢σ^Yk-2Σk⁢β^Xk 2⁢σ^Yk-2

σ^IVW=1⁢/⁢Σk⁢β^Xk 2⁢σ^Yk-2

Inverse variance weighted estimates requires all instrumental variants to be valid and would be biased if average pleiotropic effects deviated from zero. Hence, robust analyses under weaker assumptions are required to provide valid causal inferences and to assess the sensitivity across these findings. Three complementary MR methods were adopted, MR-pleiotropy residual sum and outlier (MR-PRESSO), weighted median estimator and MR-Egger regression. MR-PRESSO (Verbanck et al., 2018) takes into account the horizontal pleiotropy and gives a causal estimate corrected for it, should instrumental variables with horizontal pleiotropy be identified via MR-PRESSO global test. Weighted median (Bowden et al., 2016) yields a pooled effect size more robustly if more than 50% of instrumental variables are valid (majority valid assumption). It is not as sensitively influenced by the presence of a handful of pleiotropic variants as the IVW method. MR-Egger regression (Bowden et al., 2015) has a lower statistical power with a wide range of causality estimates. It requires pleiotropic effects to be independent of the variant–exposure associations (instrument strength independent of direct effect assumption). MR-Egger method gives an causal estimate with the regression slope, meanwhile MR-Egger intercept also provides an assessment of unbalanced horizontal pleiotropy across all variants. Additional sensitivity analyses for heterogeneity detection were performed through Cochran’s *Q* test, leave-one-out plots and funnel plots. Power calculations were conducted in *mRnd*, a web-based application (Brion et al., 2013) assuming a 80% power and 5% Type-I error rate. Statistical significance was set at 0.025 (*P* = 0.05/2 association tests) with the Bonferroni method to correct for multiple testing.

## Results

### Estimated Causal Effect of PCOS on Periodontitis

**FIGURE 2:**
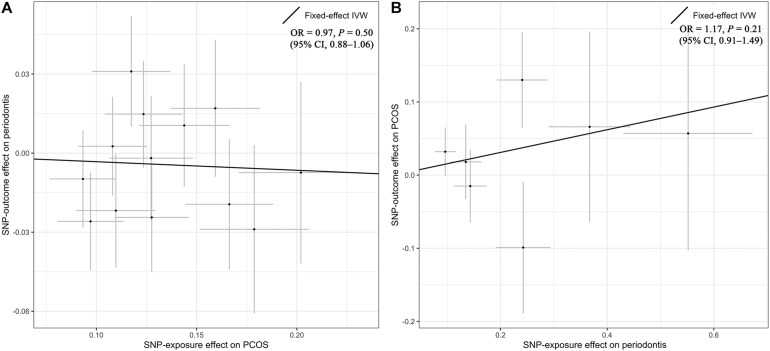
Primary results by Mendelian randomization analysis using the inverse-variance-weighted method. The causal estimate (overall fitted line) for the effect of PCOS on periodontitis was shown in panel **(A)**, while the overall effect for the casual association of periodontitis with PCOS was presented in panel **(B)**. Individual SNP-effect on the outcome (point and vertical line) against its effect on the exposure (point and horizontal line) was delineated in the background. CI, confidence interval; IVW, inverse variance weighted; OR, odds ratio; PCOS, polycystic ovary syndrome; SNP, single nucleotide polymorphism.

**FIGURE 3:**
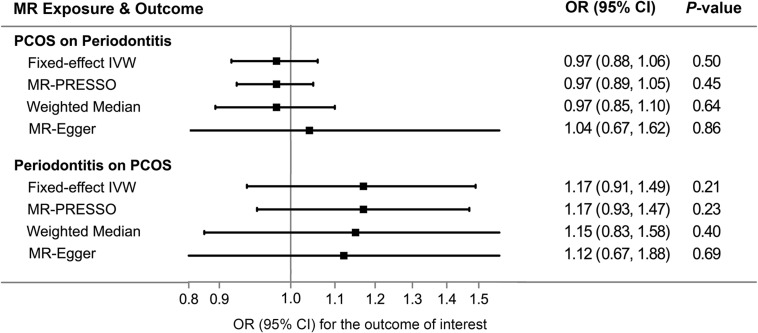
Comparisons of Mendelian randomization results by different methods. CI, confidence interval; IVW, inverse variance weighted; MR, Mendelian randomization; OR, odds ratio; PCOS, polycystic ovary syndrome; PRESSO, pleiotropy residual sum and outlier.

Overall, there was no causal relationship between genetically predicted PCOS and periodontitis. Primary MR results (Figure 2) indicated that odds ratio (OR) of periodontitis is 0.97 [95% confidence interval (CI), 0.88–1.06; *P* = 0.50] per one-unit increase in log-OR of PCOS (equivalent to 2.718 fold change in the OR of PCOS) by the IVW method, and estimates by MR-PRESSO and weighted median methods (Figure 3) were consistent with respect to the effect size and direction. Causal estimates given by MR-Egger (OR = 1.04 per one-unit increase in log-OR of PCOS) with wide 95% CI (0.67–1.62) were less precise. No horizontal pleiotropy was identified (Supplementary Table 7), as shown by MR-Egger test (Intercept = -0.009; *P* = 0.75) and MR-PRESSO global test (RSS_*obs*_ = 10.62; *P* = 0.69). There was no significant heterogeneity detected through Cochran’s *Q* test (*Q* = 9.07; *P* = 0.70). Elimination of single instrumental SNP would not lead to distortion of the overall MR estimate (Supplementary Figure 1), whereas overall symmetry of the funnel plot further demonstrated negligible heterogeneity and validated the robustness of the causal estimate given by the fixed-effect IVW method. Since the closer an OR approaching 1, the much larger a sample size is required to detect such a weak effect, this study was underpowered to detect an OR interval of 0.88–1.13 according to our power calculation.

### Estimated Causal Effect of Periodontitis on PCOS

Genetic liability to periodontitis was not causally associated with risk of PCOS. By the IVW method (Figure 2), the OR of PCOS was 1.17 (95% CI 0.91–1.49; *P* = 0.21) per one-unit increment in the log-OR of periodontitis. Causal estimates by three additional approaches (Figure 3) did not reach nominal significance, either. There was no evidence for the existence of outlier SNPs, heterogeneity or horizontal pleiotropy by the MR sensitivity analyses (Supplementary Table 7). Examination of the leave-one-out plot and funnel plot (Supplementary Figure 1) suggested that the MR results were not driven by certain SNP alone and overall causal estimates were consistent and accurate on the whole. This study was underpowered to detect an OR interval of 0.70–1.26 according to our power calculation.

## Discussion

This study explored the bidirectional relationships between PCOS and periodontitis using a two-sample MR design for the first time. The MR analysis failed to identify a causal effect of PCOS on periodontitis in European women, and the statistical power was adequate if the observed effect (OR = 1.28) in the recent meta-analysis (Machado et al., 2020) represented a true causality. Meanwhile, our results provided no evidence that genetically predicted risk of periodontitis was causally associated with liability to PCOS. Previous established relationship between predisposition to PCOS and periodontitis might result from uncontrolled biases or cofounders in observational epidemiological studies.

Two systematic reviews (Kellesarian et al., 2017; Marquez-Arrico et al., 2020) have been conducted to address the hypothesis: whether a causal relationship exists between PCOS and periodontal diseases. The qualitative evidence suggested that a variety of periodontal parameters, such as PPD and bleeding on probing, together with altered immunoinflammatory and microbiological outcomes were observed in patients with PCOS. A positive association between PCOS and periodontal diseases was concluded. However, most included studies featured the case-control design, a small sample size ranging from 52 to 196, and non-follow-up. The strength of evidence should be taken into account before promoting regular referral of PCOS patients to oral-health evaluation. Machado et al. (2020) conducted a quantitative synthesis and yielded an OR of 1.28 (95% CI 1.06–1.55; *P* < 0.001) for the effect of PCOS on periodontitis and an OR of 1.46 (95% CI 1.29–1.66; *P* < 0.001) for the reverse association. Notably, the effect estimates were derived from three Asian cohorts (Porwal et al., 2014; Tong et al., 2019; Saljoughi et al., 2020) in the meta-analysis. Discrepancies between this MR study and the recent meta-analysis in the assessment of bidirectional association might be partly explained by the population difference.

It has been postulated that PCOS and periodontitis is linked by systemic inflammation and oxidative status (Dursun et al., 2011; Saglam et al., 2018). Myeloperoxidase and nitric oxide, indicative of oxidative stress, were higher in women with PCOS than healthy controls (Marquez-Arrico et al., 2020). Likewise, increased levels of malondialdehyde and 8-hydroxy-2′-deoxyguanosine were identified in PCOS, which were prominent both in serum and gingival crevicular fluid (Saglam et al., 2018). Neutrophils are recognized to play a key role in the initiation of inflammatory responses to periodontal pathogens, and local oxidative stress is strengthened in periodontitis (Porwal et al., 2014). Hence, altered oxidative status in PCOS might contribute to the occurrence or progression of periodontitis. Considering the observed links between periodontitis and multiple diseases (Czesnikiewicz-Guzik et al., 2019; Bae and Lee, 2020; Sun et al., 2020), inflammatory response cascade in periodontitis might as well exert an influence on the risk of PCOS through molecular changes in the metabolic-endocrine networks.

Notably, PCOS is known as a heterogeneous disorder with three principal components, OD, HA, and PCOM. Several other diagnostic criteria have been adopted as well. To enhance the statistical power, there is a trade-off between phenotypic refinement and incorporating sufficiently large cohorts. However, Day et al. (2018) has demonstrated minimal heterogeneity of the SNP effect, except one SNP near GATA4/NEIL2 (rs804279, *P_*het*_* = 2.6 × 10^–5^), across NIH, Rotterdam, and self-reported criteria. Therefore, we deemed that negligible bias should be incurred when utilizing summary statistics derived from multiple cohorts. Besides, with currently available summary-level statistics, we could not perform a comprehensive MR analysis exploring the effects of different subtypes of PCOS on periodontitis. Likewise, a broad spectrum of periodontal diseases, incorporating gingivitis, moderate chronic periodontitis, and severe aggressive periodontitis, could not be considered in the MR analysis.

There are several limitations in this study. Firstly, instrumental SNPs selected from GWAS were mainly based on a statistically driven hypotheses, and genome-wide significance alone cannot guarantee the plausibility of these variants. Especially, their biological implication and complexity has not been fully understood, let alone thoroughly examined. Notably, seven instrumental SNPs for periodontitis were only suggestively significant (*P* < 5 × 10^–6^). Therefore, we should be cautious with the null effect in the MR analysis of periodontitis on PCOS, which might be due to the lack of association strength of instrumental SNPs. Secondly, current GWAS design and analysis models cannot take all sources of potential bias into account, such as Collider bias, winner’s curse and Beavis effects. Given that the genetic estimates of SNP-association underlie the further MR analysis, there might be bias incurred into the MR causal estimates as well. Thirdly, selected instrumental variants collectively explained a small proportion of variance of PCOS or periodontitis. Thus, we were not capable of detecting weak effects, although horizontal pleiotropy and weak instrument bias have been ruled out. Forthly, with summary-level data, we failed to conduct a stratified analysis exploring the effects of PCOS based on body mass index or obesity status, which has been proposed to account for diverged outcomes in PCOS. Moreover, GWAS estimates of PCOS were from studies in women while the corresponding estimates of periodontitis were not restricted to female participants, which could possibly introduce bias. Lastly, data sets were of European ancestry, and cautions should be exercised when interpreting and generalizing the MR results.

To conclude, this bidirectional MR study failed to provide convincing evidence to support the causal relationshipbetween genetic liability to PCOS and periodontitis. To elucidate previously observed links, high-qualified clinical trials and laboratory researches are warranted. Triangulation of evidence across multiple study designs is essential when assessing the association between PCOS and periodontitis.

## Data Availability Statement

The original contributions presented in the study are included in the article/Supplementary Material, further inquiries can be directed to the corresponding author.

## Author Contributions

PW, XZ, and PZ conceptualized the study. PW, XZ, WZ, DL, and ML took part in the data curation, methodology, software, and formal analysis. PW, XZ, PZ, ML, and XL were in charge of the validation and visualization. All authors contributed to the article and approved the submitted version.

## Conflict of Interest

The authors declare that the research was conducted in the absence of any commercial or financial relationships that could be construed as a potential conflict of interest.

## Footnotes

1 https://www.repository.cam.ac.uk/handle/1810/289950

2 https://data.bris.ac.uk/data/dataset/2j2rqgzedxlq02oqbb4vmycnc2

## References

[B1] Azziz R. Kintziger K. Li R. Laven J. Morin-Papunen L. Merkin S. S. (2019). Recommendations for epidemiologic and phenotypic research in polycystic ovary syndrome: an androgen excess and PCOS society resource. *Hum. Reprod.* 34 2254–2265. 10.1093/humrep/dez185 31751476

[B2] Bae S. C. Lee Y. H. (2020). Causal association between periodontitis and risk of rheumatoid arthritis and systemic lupus erythematosus: a Mendelian randomization. *Z. Rheumatol.* 79 929–936. 10.1007/s00393-019-00742-w 31965238

[B3] Bowden J. Davey Smith G. Burgess S. (2015). Mendelian randomization with invalid instruments: effect estimation and bias detection through Egger regression. *Int. J. Epidemiol.* 44 512–525. 10.1093/ije/dyv080 26050253 PMC4469799

[B4] Bowden J. Davey Smith G. Haycock P. C. Burgess S. (2016). Consistent estimation in mendelian randomization with some invalid instruments using a weighted median estimator. *Genet. Epidemiol.* 40 304–314. 10.1002/gepi.21965 27061298 PMC4849733

[B5] Brion M. J. Shakhbazov K. Visscher P. M. (2013). Calculating statistical power in Mendelian randomization studies. *Int. J. Epidemiol.* 42 1497–1501. 10.1093/ije/dyt179 24159078 PMC3807619

[B6] Buniello A. MacArthur J. A. L. Cerezo M. Harris L. W. Hayhurst J. Malangone C. (2019). The NHGRI-EBI GWAS catalog of published genome-wide association studies, targeted arrays and summary statistics 2019. *Nucleic Acids Res.* 47 D1005–D1012. 10.1093/nar/gky1120 30445434 PMC6323933

[B7] Burgess S. Butterworth A. Thompson S. G. (2013). Mendelian randomization analysis with multiple genetic variants using summarized data. *Genet. Epidemiol.* 37 658–665. 10.1002/gepi.21758 24114802 PMC4377079

[B8] Burgess S. Davey Smith G. Davies N. M. Dudbridge F. Gill D. Glymour M. M. (2019). Guidelines for performing Mendelian randomization investigations. *Wellcome Open Res.* 4 186. 10.12688/wellcomeopenres.15555.1 PMC7384151 32760811

[B9] Carmina E. (2004). Diagnosis of polycystic ovary syndrome: from NIH criteria to ESHRE-ASRM guidelines. *Minerva Ginecol.* 56 1–6. 14973405

[B10] Chaffee B. W. Persai D. Vora M. V. (2020). Interdental cleaning and oral health status in an adult cohort, 2015 to 2018. *J. Dent. Res.* 99 1150–1156. 10.1177/0022034520926139 32464077 PMC7443997

[B11] Czesnikiewicz-Guzik M. Osmenda G. Siedlinski M. Nosalski R. Pelka P. Nowakowski D. (2019). Causal association between periodontitis and hypertension: evidence from Mendelian randomization and a randomized controlled trial of non-surgical periodontal therapy. *Eur. Heart J.* 40 3459–3470. 10.1093/eurheartj/ehz646 31504461 PMC6837161

[B12] Davies N. M. Holmes M. V. Davey Smith G. (2018). Reading Mendelian randomisation studies: a guide, glossary, and checklist for clinicians. *BMJ* 362:k601. 10.1136/bmj.k601 30002074 PMC6041728

[B13] Day F. Karaderi T. Jones M. R. Meun C. He C. Drong A. (2018). Large-scale genome-wide meta-analysis of polycystic ovary syndrome suggests shared genetic architecture for different diagnosis criteria. *PLoS Genet.* 14:e1007813. 10.1371/journal.pgen.1007813 30566500 PMC6300389

[B14] Day F. Karaderi T. Jones M. R. Meun C. He C. Drong A. (2019). Correction: large-scale genome-wide meta-analysis of polycystic ovary syndrome suggests shared genetic architecture for different diagnosis criteria. *PLoS Genet.* 15:e1008517. 10.1371/journal.pgen.1008517 31805045 PMC6894746

[B15] Day F. R. Hinds D. A. Tung J. Y. Stolk L. Styrkarsdottir U. Saxena R. (2015). Causal mechanisms and balancing selection inferred from genetic associations with polycystic ovary syndrome. *Nat. Commun.* 6:8464. 10.1038/ncomms9464 26416764 PMC4598835

[B16] Divaris K. Monda K. L. North K. E. Olshan A. F. Reynolds L. M. Hsueh W. C. (2013). Exploring the genetic basis of chronic periodontitis: a genome-wide association study. *Hum. Mol. Genet.* 22 2312–2324. 10.1093/hmg/ddt065 23459936 PMC3652417

[B17] Dursun E. Akalin F. A. Guncu G. N. Cinar N. Aksoy D. Y. Tozum T. F. (2011). Periodontal disease in polycystic ovary syndrome. *Fertil. Steril.* 95 320–323. 10.1016/j.fertnstert.2010.07.1052 20800834

[B18] Eke P. I. Borgnakke W. S. Genco R. J. (2020). Recent epidemiologic trends in periodontitis in the USA. *Periodontology* 2000 257–267. 10.1111/prd.12323 31850640

[B19] Escobar-Morreale H. F. (2018). Polycystic ovary syndrome: definition, aetiology, diagnosis and treatment. *Nat. Rev. Endocrinol.* 14 270–284. 10.1038/nrendo.2018.24 29569621

[B20] Hemani G. Zheng J. Elsworth B. Wade K. H. Haberland V. Baird D. (2018). The MR-Base platform supports systematic causal inference across the human phenome. *Elife* 7:e34408. 10.7554/eLife.34408 29846171 PMC5976434

[B21] Isik Y. Telatar G. Y. Neselioglu S. Bicer C. Gurlek B. (2020). Evaluation of periodontal status in different phenotypes of polycystic ovary syndrome in untreated patients of early reproductive age: a case-control study. *J. Obstet. Gynaecol. Res.* 46 459–465. 10.1111/jog.14179 31922343

[B22] Kellesarian S. V. Malignaggi V. R. Kellesarian T. V. Al-Kheraif A. A. Alwageet M. M. Malmstrom H. (2017). Association between periodontal disease and polycystic ovary syndrome: a systematic review. *Int. J. Impot. Res.* 29 89–95. 10.1038/ijir.2017.7 28275229

[B23] Kurushima Y. Tsai P. C. Castillo-Fernandez J. Couto Alves A. El-Sayed Moustafa J. S. Le Roy C. (2019). Epigenetic findings in periodontitis in UK twins: a cross-sectional study. *Clin. Epigenetics* 11:27. 10.1186/s13148-019-0614-4 30760334 PMC6375219

[B24] Machado V. Escalda C. Proenca L. Mendes J. J. Botelho J. (2020). Is there a bidirectional association between polycystic ovarian syndrome and periodontitis? A systematic review and meta-analysis. *J. Clin. Med.* 9:1961. 10.3390/jcm9061961 32585861 PMC7355910

[B25] Marquez-Arrico C. F. Silvestre-Rangil J. Gutierrez-Castillo L. Martinez-Herrera M. Silvestre F. J. Rocha M. (2020). Association between periodontal diseases and polycystic ovary syndrome: a systematic review. *J. Clin. Med.* 9:1586. 10.3390/jcm9051586 32456146 PMC7290429

[B26] Munz M. Richter G. M. Loos B. G. Jepsen S. Divaris K. Offenbacher S. (2019). Meta-analysis of genome-wide association studies of aggressive and chronic periodontitis identifies two novel risk loci. *Eur. J. Hum. Genet.* 27 102–113. 10.1038/s41431-018-0265-5 30218097 PMC6303247

[B27] Munz M. Willenborg C. Richter G. M. Jockel-Schneider Y. Graetz C. Staufenbiel I. (2017). A genome-wide association study identifies nucleotide variants at SIGLEC5 and DEFA1A3 as risk loci for periodontitis. *Hum. Mol. Genet.* 26 2577–2588. 10.1093/hmg/ddx151 28449029

[B28] Myers T. A. Chanock S. J. Machiela M. J. (2020). LDlinkR: an R Package for rapidly calculating linkage disequilibrium statistics in diverse populations. *Front. Genet.* 11:157. 10.3389/fgene.2020.00157 32180801 PMC7059597

[B29] Offenbacher S. Divaris K. Barros S. P. Moss K. L. Marchesan J. T. Morelli T. (2016). Genome-wide association study of biologically informed periodontal complex traits offers novel insights into the genetic basis of periodontal disease. *Hum. Mol. Genet.* 25 2113–2129. 10.1093/hmg/ddw069 26962152 PMC5062586

[B30] Page R. C. Eke P. I. (2007). Case definitions for use in population-based surveillance of periodontitis. *J. Periodontol.* 78(7 Suppl.) 1387–1399. 10.1902/jop.2007.060264 17608611

[B31] Porwal S. Tewari S. Sharma R. K. Singhal S. R. Narula S. C. (2014). Periodontal status and high-sensitivity C-reactive protein levels in polycystic ovary syndrome with and without medical treatment. *J. Periodontol.* 85 1380–1389. 10.1902/jop.2014.130756 24592911

[B32] Rotterdam ESHRE Group (2004). Revised 2003 consensus on diagnostic criteria and long-term health risks related to polycystic ovary syndrome (PCOS). *Hum. Reprod.* 19 41–47. 10.1093/humrep/deh098 14688154

[B33] Saglam E. Canakci C. F. Sebin S. O. Saruhan N. Ingec M. Canakci H. (2018). Evaluation of oxidative status in patients with chronic periodontitis and polycystic ovary syndrome: a cross-sectional study. *J. Periodontol.* 89 76–84. 10.1902/jop.2017.170129 28844187

[B34] Saljoughi F. Nasri K. Bayani M. (2020). Gingival crevicular fluid levels of visfatin in patients with chronic periodontitis and polycystic ovary syndrome. *Obstet. Gynecol. Sci.* 63 87–93. 10.5468/ogs.2020.63.1.87 31970132 PMC6962584

[B35] Shungin D. Haworth S. Divaris K. Agler C. S. Kamatani Y. Keun Lee M. (2019). Genome-wide analysis of dental caries and periodontitis combining clinical and self-reported data. *Nat. Commun.* 10:2773. 10.1038/s41467-019-10630-1 31235808 PMC6591304

[B36] Sirmans S. M. Pate K. A. (2013). Epidemiology, diagnosis, and management of polycystic ovary syndrome. *Clin. Epidemiol.* 6 1–13. 10.2147/CLEP.S37559 24379699 PMC3872139

[B37] Sun Y. Q. Richmond R. C. Chen Y. Mai X. M. (2020). Mixed evidence for the relationship between periodontitis and Alzheimer’s disease: a bidirectional mendelian randomization study. *PLoS One* 15:e0228206. 10.1371/journal.pone.0228206 31978120 PMC6980529

[B38] Tong C. Wang Y. H. Yu H. C. Chang Y. C. (2019). Increased risk of polycystic ovary syndrome in taiwanese women with chronic periodontitis: a nationwide population-based retrospective cohort study. *J. Womens Health (Larchmt)* 28 1436–1441. 10.1089/jwh.2018.7648 31145020

[B39] Verbanck M. Chen C. Y. Neale B. Do R. (2018). Detection of widespread horizontal pleiotropy in causal relationships inferred from Mendelian randomization between complex traits and diseases. *Nat. Genet.* 50 693–698. 10.1038/s41588-018-0099-7 29686387 PMC6083837

[B40] Yu Y. H. Doucette-Stamm L. Rogus J. Moss K. Zee R. Y. L. Steffensen B. (2018). Family history of MI, smoking, and risk of periodontal disease. *J. Dent. Res.* 97 1106–1113. 10.1177/0022034518782189 29928831 PMC6169032

