# Supplementary material for: Assessment of Bidirectional Relationships Between Polycystic Ovary Syndrome and Periodontitis: Insights From a Mendelian Randomization Analysis
Source: Front Genet. 2021 Mar 26;12:644101. doi: 10.3389/fgene.2021.644101 (PMC8044848; doi:10.3389/fgene.2021.644101)
Supplement: Supplementary file 1 [file Data_Sheet_1.PDF]

Supplementary Tables and Figures

Table S1. Genetic instrumental variables utilized in the Mendelian randomization analysis of polycystic ovary syndrome on periodontitis

Table S2. Look-up of potential pleiotropic associations ($P < 5 \times 10^{-8}$) of instrumental SNPs for PCOS in the GWAS Catalog

Table S3. Assessment of genetic instrumental variables for polycystic ovary syndrome

Table S4. Genetic instrumental variables utilized in the Mendelian randomization analysis of periodontitis on polycystic ovary syndrome

Table S5. Look-up of potential pleiotropic associations ($P < 5 \times 10^{-8}$) of instrumental SNPs for periodontitis in the GWAS Catalog

Table S6. Assessment of genetic instrumental variables for periodontitis

Table S7. Results of Mendelian randomization sensitivity analyses

Figure S1. Leave-one-out plots and funnel plots in the sensitivity analyses

Table S1 Genetic instrumental variables utilized in the Mendelian randomization analysis of polycystic ovary syndrome on periodontitis

SNP	Chr:Pos	Nearest Gene	EA/OA	EAF	Association with PCOS			Association with periodontitis		
					Beta	SE	*P*-value	Beta	SE	*P*-value
rs2178575	2:213391766	ERBB4	A/G	0.15	0.166	0.022	3.34×10^{-14}	-0.019	0.025	0.43
rs11031005	11:30226356	ARL14EP/FSHB	T/C	0.85	-0.159	0.022	8.66×10^{-13}	-0.017	0.026	0.51
rs804279	8:11623889	GATA4/NEIL2	A/T	0.26	0.128	0.018	3.76×10^{-12}	-0.024	0.021	0.24
rs11225154	11:102043240	YAP1	A/G	0.09	0.179	0.027	5.44×10^{-11}	-0.029	0.032	0.37
rs9696009	9:126619233	DENND1A	A/G	0.07	0.202	0.031	7.96×10^{-11}	-0.007	0.034	0.83
rs13164856	5:131813204	IRF1/RAD50	T/C	0.73	0.124	0.019	1.45×10^{-10}	0.015	0.020	0.47
rs1784692	11:113949232	ZBTB16	T/C	0.82	0.144	0.023	1.88×10^{-10}	0.011	0.023	0.65
rs7563201	2:43561780	THADA	A/G	0.45	-0.108	0.017	3.68×10^{-10}	-0.003	0.019	0.89
rs8043701	16:52375777	TOX3	A/T	0.82	-0.127	0.021	9.61×10^{-10}	0.002	0.024	0.94
rs1795379	12:75941042	KRR1	T/C	0.24	-0.117	0.020	1.81×10^{-9}	-0.031	0.021	0.14
rs2271194	12:56477694	ERBB3/RAB5B	A/T	0.42	0.097	0.017	4.57×10^{-9}	-0.026	0.019	0.16
rs10739076	9:5440589	PLGRKT	A/C	0.31	0.110	0.020	2.51×10^{-8}	-0.022	0.022	0.31
rs7864171	9:97723266	C9orf3	A/G	0.43	-0.093	0.017	2.95×10^{-8}	0.010	0.018	0.59

Abbreviations: Beta represents one-unit change in the log-odds of developing PCOS or periodontitis per additional effect allele; Chr:Pos, Chromosome and position according to GRCh37/hg19 genome assembly; EA/OA, effect allele/reference allele; EAF, effect allele frequency; PCOS, polycystic ovary syndrome; SE, standard error; SNP, single nucleotide polymorphism.

Table S2 Look-up of potential pleiotropic associations ($P < 5 \times 10^{-8}$) of instrumental SNPs for PCOS in the GWAS Catalog

SNP	Chr	Nearest Gene	Traits and Studies
rs7563201	2	THADA	NA
rs2178575	2	ERBB4	NA
rs13164856	5	IRF1/RAD50	Asthma (PMID: 31669095)
rs804279	8	GATA4/NEIL2	NA
rs10739076	9	PLGRKT	NA
rs7864171	9	C9orf3	NA
rs9696009	9	DENND1A	NA
rs11031005	11	ARL14EP/FSHB	Testosterone (PMID: 32042192), Follicle-stimulating hormone (PMID: 26014426), Age at menopause (PMID: 30595370), Age at menarche (PMID: 27182965)
rs11225154	11	YAP1	NA
rs1784692	11	ZBTB16	NA
rs2271194	12	ERBB3/RAB5B	Autoimmune thyroid disease (PMID: 32581359)
rs1795379	12	KRR1	NA
rs8043701	16	TOX3	NA

Abbreviations: CHR, chromosome; GWAS, genome-wide association study; PCOS, polycystic ovary syndrome; SNP, Single-nucleotide polymorphism.

Table S3 Assessment of genetic instrumental variables for polycystic ovary syndrome

SNP	Chr:Pos	Effect allele	MAF	Beta	*P*-value	R^2	*F*
rs7563201	2:43561780	A	0.45	-0.11	3.68×10^{-10}	0.0058	39.5
rs2178575	2:213391766	A	0.15	0.17	3.34×10^{-14}	0.0071	57.6
rs13164856	5:131813204	T	0.27	0.12	1.45×10^{-10}	0.0060	40.9
rs804279	8:11623889	A	0.26	0.13	3.76×10^{-12}	0.0063	48.1
rs10739076	9:5440589	A	0.31	0.11	2.51×10^{-8}	0.0051	31.0
rs7864171	9:97723266	A	0.43	-0.09	2.95×10^{-8}	0.0043	30.8
rs9696009	9:126619233	A	0.07	0.20	7.96×10^{-11}	0.0052	42.2
rs11031005	11:30226356	T	0.15	-0.16	8.66×10^{-13}	0.0063	51.0
rs11225154	11:102043240	A	0.09	0.18	5.44×10^{-11}	0.0054	43.2
rs1784692	11:113949232	T	0.18	0.14	1.88×10^{-10}	0.0060	40.5
rs2271194	12:56477694	A	0.42	0.10	4.57×10^{-9}	0.0046	34.2
rs1795379	12:75941042	T	0.24	-0.12	1.81×10^{-9}	0.0050	36.2
rs8043701	16:52375777	A	0.18	-0.13	9.61×10^{-10}	0.0049	37.5

Note: Proportion of variance explained was calculated using formula**:** $\boldsymbol{R^2 = 2 \times \mathrm{MAF} \times (1\text{-}\mathrm{MAF}) \times \mathrm{Beta}^2}$, and total variance ~ 6.2% was further utilized in the power calculation (https://shiny.cnsgenomics.com/mRnd/). Strength of each SNP was assessed by ***F*-statistic =** $\boldsymbol{R^2(N-2)/(1-R^2)}$, where $F < 10$ was deemed as a weak instrument.

Abbreviations: SNP, Single-nucleotide polymorphism; MAF, minor allele frequency; R^2, proportion of variance explained; N = 113, 238 was the sample size of the GWAS of polycystic ovary syndrome.

Table S4 Genetic instrumental variables utilized in the Mendelian randomization analysis of periodontitis on polycystic ovary syndrome

SNP	Chr:Pos	Nearest Gene	EA/OA	EAF	Association with periodontitis			Association with PCOS		
					**Beta**	**SE**	***P*-value**	**Beta**	**SE**	***P*-value**
rs4956201	4:109527782	RPL34-AS1	A/C	0.89	-0.241	0.047	3.89×10^{-7}	-0.130	0.065	0.05
rs2976950	8:8249082	SGK223	A/G	0.60	0.096	0.020	7.99×10^{-7}	0.032	0.033	0.33
rs151226594	11:64256137	LOC100996455	T/G	0.01	-0.367	0.077	1.75×10^{-6}	-0.066	0.130	0.60
rs78422482	4:19970150	SLIT2	A/G	0.01	0.243	0.051	2.02×10^{-6}	-0.099	0.090	0.27
rs13005050	2:52705571	MIR4431	T/C	0.14	-0.143	0.031	3.76×10^{-6}	0.015	0.050	0.76
rs6816769	4:122216017	QRFPR	T/C	0.89	-0.135	0.029	4.57×10^{-6}	-0.018	0.051	0.73
rs117710629	18:39372393	PIK3C3	A/G	0.01	-0.552	0.121	4.96×10^{-6}	-0.057	0.160	0.72

Abbreviations: Beta represents one-unit change in the log-odds of developing PCOS or periodontitis per additional effect allele; Chr:Pos, Chromosome and position according to GRCh37/hg19 genome assembly; EA/OA, effect allele/reference allele; EAF, effect allele frequency; PCOS, polycystic ovary syndrome; SE, standard error; SNP, single nucleotide polymorphism.

Table S5 Look-up of potential pleiotropic associations ($P < 5 \times 10^{-8}$) of instrumental SNPs for periodontitis in the GWAS Catalog

SNP	Chr	Nearest Gene	Traits and Studies
rs13005050	2	MIR4431	NA
rs78422482	4	SLIT2	NA
rs4956201	4	RPL34-AS1	NA
rs6816769	4	QRFPR	NA
rs2976950	8	SGK223	NA
rs151226594	11	LOC100996455	NA
rs117710629	18	PIK3C3	NA

Abbreviations: CHR, chromosome; GWAS, genome-wide association study; PCOS, polycystic ovary syndrome; SNP, Single-nucleotide polymorphism.

Table S6 Assessment of genetic instrumental variables for periodontitis

SNP	Chr:Pos	Effect allele	MAF	Beta	*P*-value	R^2	*F*
rs13005050	2:52705571	T	0.14	-0.14	3.76×10^{-6}	0.0049	171.3
rs78422482	4:19970150	A	0.01	0.24	2.02×10^{-6}	0.0012	40.5
rs4956201	4:109527782	A	0.11	-0.24	3.89×10^{-7}	0.0114	398.2
rs6816769	4:122216017	T	0.11	-0.14	4.57×10^{-6}	0.0036	124.0
rs2976950	8:8249082	A	0.40	0.10	7.99×10^{-7}	0.0044	153.8
rs151226594	11:64256137	T	0.01	-0.37	1.75×10^{-6}	0.0027	92.6
rs117710629	18:39372393	A	0.01	-0.55	4.96×10^{-6}	0.0050	172.4

Note: Proportion of variance explained was calculated using formula**:** $\boldsymbol{R^2 = 2 \times \text{MAF} \times (1\text{-MAF}) \times \text{Beta}^2}$, and total variance ~ 3.3% was further utilized in the power calculation (https://shiny.cnsgenomics.com/mRnd/). Strength of each SNP was assessed by $\boldsymbol{F\text{-statistic} = R^2(\text{N}-2)/(1-R^2)}$, where $F < 10$ was deemed as a weak instrument. MAF, was from the 1000Genomes European panel, since allele frequency variable has been removed to prevent re-identification of individuals in the shared summary-level dataset of periodontitis.

Abbreviations: SNP, Single-nucleotide polymorphism; MAF, minor allele frequency; R^2, proportion of variance explained; N = 34, 615 was the sample size of the GWAS of periodontitis.

Table S7 Results of Mendelian randomization sensitivity analyses

MR analyses	MR-Egger regression		MR-PRESSO global test		Cochran's *Q* test	
	Intercept	*P*-value	RSS_{obs}	*P*-value	*Q*-statistic	*P*-value
PCOS on periodontitis	-0.009	0.75	10.62	0.69	9.07	0.70
Periodontitis on PCOS	0.008	0.86	7.34	0.50	5.20	0.52

Abbreviations: MR, Mendelian randomization; PRESSO, Pleiotropy RESidual Sum and Outlier model; PCOS, polycystic ovary syndrome; RSS_{obs}, observed residual sum of squares

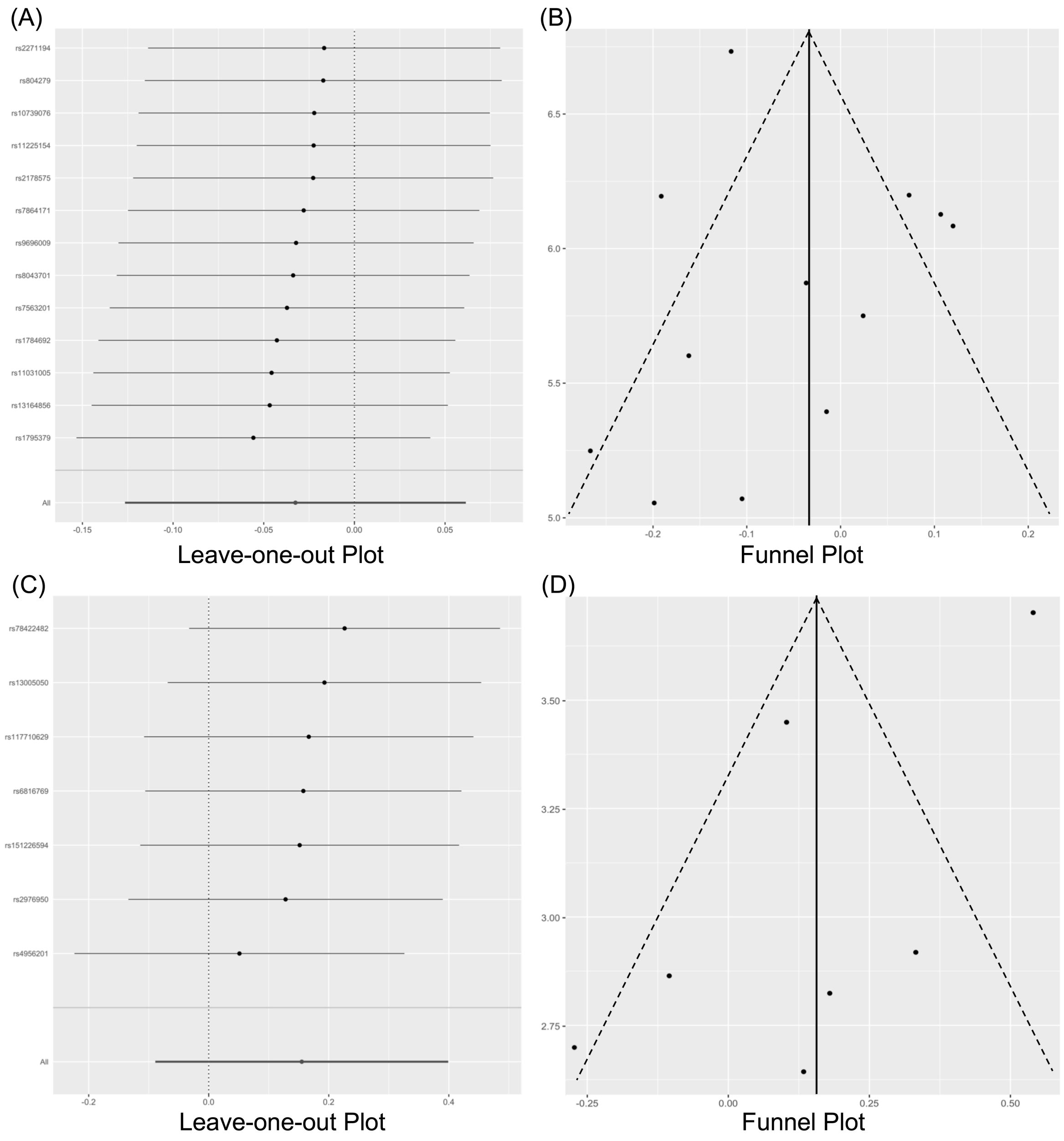

Figure S1. Leave-one-out plots and funnel plots in the sensitivity analyses. The leave-one-out plot (**A**) visualized how the causal estimates (point with horizontal line) for the effect of polycystic ovary syndrome (PCOS) on periodontitis were influenced by the removal of single variant. The funnel plot (**B**) illustrated the overall symmetry of causal estimates across all instrumental variables. Each point denoted corresponding inverse standard error against the individual causal estimate. The leave-one-out plot (**C**) and funnel plot (**D**) in the sensitivity analysis of the periodontitis-PCOS relationship were presented likewise.
